# Shizukaol D Isolated from *Chloranthus* *japonicas* Inhibits AMPK-Dependent Lipid Content in Hepatic Cells by Inducing Mitochondrial Dysfunction

**DOI:** 10.1371/journal.pone.0073527

**Published:** 2013-08-14

**Authors:** Rongkuan Hu, Huan Yan, Xiaojiang Hao, Haiyang Liu, Jiarui Wu

**Affiliations:** 1 Hefei National Laboratory for Physical Sciences at Microscale and School of Life Sciences, University of Science & Technology of China, Hefei, Anhui, the People’s Republic of China; 2 State Key Laboratory of Phytochemistry and Plant Resources in West China, Kunming Institute of Botany, Chinese Academy of Sciences, Kunming, Yunnan, the People’s Republic of China; 3 Key Laboratory of Systems Biology, Institute of Biochemistry and Cell Biology, Shanghai Institutes for Biological Sciences, Chinese Academy of Sciences, Shanghai, the People’s Republic of China; 4 Shanghai Advanced Research Institute, Chinese Academy of Sciences, Shanghai, the People’s Republic of China; Broad Institute of Harvard and MIT, United States of America

## Abstract

This study is the first to demonstrate that shizukaol D, a natural compound isolated from *Chloranthus* *japonicus* , can activate AMP- activated protein kinase (AMPK), a key sensor and regulator of intracellular energy metabolism, leading to a decrease in triglyceride and cholesterol levels in HepG2 cells. Furthermore, we found that shizukaol D induces mitochondrial dysfunction by depolarizing the mitochondrial membrane and suppressing energy production, which may result in AMPK activation. Our results provide a possible link between mitochondrial dysfunction and AMPK activation and suggest that shizukaol D might be used to treat metabolic syndrome.

## Introduction

AMPK is an efficient sensor of cellular energy states and is a downstream target of many kinases [1–3]. It is activated in response to a variety of metabolic stresses such as hypoxia and nutrient deprivation [4–7]. Once AMPK is activated, it orchestrates a variety of metabolic processes to increase ATP production and to decrease ATP consumption [8–10]. AMPK activation results in the phosphorylation of acetyl-CoA carboxylase (ACC), a direct AMPK substrate, at Ser 79 [11–13], leading to decreased conversion from acetyl-CoA to malonyl-CoA, which is important for fatty acid synthesis [14,15]. AMPK activation also results in the phosphorylation and activation of malonyl-CoA decarboxylase (MCD), which leads to a further decrease in malonyl-CoA levels [16,17]. Malonyl-CoA inhibits carnitine-palmitoyl-CoA transferase 1 (CPT 1), an enzyme responsible for transporting long-chain fatty acids into mitochondria to be oxidized [17,18]. Overall, AMPK activation decreases fatty acid synthesis and induces fatty acid oxidation, leading to decreased lipid accumulation *in vitro* and *in vivo* [8,19].

A number of anti-diabetic drugs such as metformin and the thiazolidinediones (TZDs) regulate AMPK activity [20,21]. Metformin increases AMPK phosphorylation and mediates fatty acid oxidation and synthesis [22,23]. Thiazolidinediones increase the cellular AMP/ATP ratio, which leads to AMPK activation [24,25]. In addition, several natural products with reported anti-obesity or anti-diabetes properties also affect AMPK activation. For example, arctigenin activates AMPK via the inhibition of mitochondria complex I and ameliorates metabolic disorders in ob/ob mice [26], and the small molecule A-769662 activates AMPK and ameliorates metabolic syndrome in ob/ob mice [27].

Given the importance of AMPK in metabolic disorders [8,14], we conducted a systematical analysis for AMPK activation in HepG2 cells treated with natural compounds isolated from *Chloranthus* *japonicus* *.* *Chloranthus* *japonicus* (Chloranthaceae) is widely used in traditional Chinese medicine for the treatment of traumatic injuries, rheumatic arthralgia, bone fractures, pulmonary tuberculosis, and neurasthenia [28,29]. The main chemical components of this plant are sesquiterpenoid dimers and trimers [30–32]. Lindenane sesquiterpenoids and disesquiterpenoids are chemotaxonomic characteristics of *Chloranthus* species. These terpenoids are derived from the enzymatic Diels-Alder cycloaddition of two lindenane-type sesquiterpenoids forming C-15-C-9' and C-6－C-8' linkages based on the *cis* and *endo* rules. This class of highly complex compounds exhibits a wide spectrum of biological activities. The disesquiterpenoids shizukaol B, shizukaol F, and cycloshizukaol A inhibit the expression of cell adhesion molecules [33], and shizukaol B, shizukaol C, shizukaol F, and shizukaol H exhibit anti-HIV activity [34]. In addition, shizukaol D shows significant anti-inflammatory activity [35].

Our results show that shizukaol D, which has not been previously shown to have metabolic activities, activates AMPK and reduces the lipid content in HepG2 cells via an AMPK-dependent mechanism. We further show that the activation of AMPK by shizukaol D may be caused by mitochondrial dysfunction.

## Materials and Methods

### Materials

1, 1-dimethylbiguanide (metformin); 5-aminoimidazole-4-carboxamide-1-D-ribofurano-side (AICAR); 5,5’, 6,6’-tetrachloro-1; 1’, 3,3’-tetraethyl-imidacarbocyanine iodide (JC-1); carbonyl cyanide m-chlorophenylhydrazone (CCCP); rosiglitazone; adenosine 5’-triphosphate (ATP) disodium salt hydrate; adenosine 5’-diphosphate sodium salt (ADP); 8-bromoadenosine 3’,5’-cyclic monophosphate (AMP); the mitochondria isolation kit for profiling cultured cells; Free Glycerol Reagent; and Triglyceride Reagent were purchased from Sigma Aldrich (St. Louis, MO, USA). 6-(4-(2-piperidin-1-ylethoxy) phenyl)-3-pyridin-4-ylpyrazolo (1, 5-a) pyrimidine (compound C) was purchased from Merck Millipore (Darmstadt, Germany). LabAssay Triglyceride and LabAssay Cholesterol kits were purchased from Wako, Japan. Antibodies against AMPKa, AMPKa1, phospho-AMPKa (Thr172), Acetyl-CoA Carboxylase (ACC), phospho-ACC (Ser79) were purchased from Cell Signaling Technology (Beverly, MA, USA). AMPKa1 siRNA and RNiMAX were purchased from Ambion, Life Technologies (NY, USA). Free fatty acids quantification kit was purchased from Biovision (CA, USA). The RIPA buffer, Bradford protein assay kit, and MTT cell proliferation and cytotoxicity assay kit were obtained from the Beyotime Institute of Biotechnology (JiangSu, China). The lactate assay kit was obtained from the Nanjing Jiancheng Bioengineering Institute (JiangSu, China).

### Shizukaol D Preparation and Structural Identification

*Chloranthus* *japonicus* is widely distributed in eastern Asia, including mainland China, Korea, and Japan, and is not an endangered or protected species in China. Furthermore, this plant is used in traditional medicine to treat traumatic injuries, rheumatic arthralgia, fractures, pulmonary tuberculosis, and neurasthenia. The plant materials in our experiment were purchased from the Chinese medicinal material market in Panshi, Jilin Province, China. The air-dried and powdered *Chloranthus* *japonicus* plants (10 kg) were extracted three times with 95% EtOH (3 × 40 L) under reflux conditions. The filtrate was evaporated under reduced pressure, yielding a residue (740 g) that was dissolved in H_2_O and extracted with AcOEt and then *n*-BuOH. The AcOEt extract (380 g) was passed through a MCI gel CHP20P column and eluted with a MeOH-H_2_O gradient (3:7 → 5:5 → 7:3 → 1: 0). The 70% MeOH fraction (110 g) was subjected to chromatography over a silica gel column (CHCl_3_-MeOH, 100:1 → 80:1 → 60:1 → 40:1) to yield six fractions, A-F. Fraction C (20 g) was separated on an Rp-18 column and eluted with a MeOH-H_2_O gradient (35%, 40%, 45%, 50%, and 55%) to obtain eight fractions, C_1_–C_8_. Fraction C_7_ was separated by silica gel column chromatography (CHCl_3_-MeOH, 100:1 → 80:1 → 60:1) and then purified on a Sephadex LH-20 (MeOH) column to yield shizukaol D (20 mg; yield: 0.0002%; purity > 98%). The structure of the purified shizukaol D was confirmed by electron spray mass spectrometry (ESIMS) and ^1^H and ^13^C-NMR spectrometry: ESI-MS *m/z*: 601 [M+Na]^+^ (C_33_H_38_O_9_) (Table 1).

**Table 1:** NMR data for shizukaol D.

Position	δ_H_	δ_C_	Position	δ_H_	δ_C_
1	2.06 (m)	25.6 (d)	3'	1.10 (m)	21.7 (d)
2*α*	1.0 (m)	15.8 (t)	4'	1.58 (dd, *J* = 13.4, 5.6 Hz)	42.9 (d)
2*β*	0.30 (m)		5'	1.83 (m)	59.1 (d)
3	1.86 (m)	24.7 (d)	6'*α*	2.45 (m)	25.0 (t)
4		142.4 (s)	6'*β*	2.47 (m)	
5		131.6 (s)	7'		168.6 (s)
6	3.91 (d, *J* = 3.5 Hz)	40.6 (d)	8'		93.3 (s)
7		131.6 (s)	9'	1.92 (dd, *J* = 5.9, 1.5 Hz)	54.5 (d)
8		200.6 (s)	10'		44.0 (s)
9	4.06 (s)	79.9 (d)	11'		126.6 (s)
10		51.0 (s)	12'		172.4 (s)
11		147.1 (s)	13'*α*	4.33 (d, *J* = 13.6 Hz)	54.9 (t)
12		171.0 (s)	13'*β*	4.39 (d, *J* = 13.6 Hz	
13	1.90 (s)	20.5 (q)	14'	0.66 (s)	24.0 (q)
14	1.02 (s)	15.3 (q)	15'*α*	3.78 (dd, *J* = 11.5, 8.3 Hz)	66.2 (t)
15*α*	2.77 (dd, *J* = 16.2, 1.5 Hz)	25.5 (t)	15'*β*	3.98 (dd, *J* = 11.5, 6.5 Hz)	
15*β*	2.61 (m)		CH_3_CO	2.08 (s)	20.8 (q)
1'	1.45 (m)	24.3 (d)	CH_3_CO		171.1 (s)
2'*α*	0.77 (m)	16.6 (t)	OMe	3.79 (s)	52.7 (q)
2'*β*	0.83 (m)				

### Cell culture

HepG2 cells (American Type Culture Collection, Manassas, VA, USA) were cultured in Dulbecco’s modified Eagle medium (DMEM) (GIBCO, Life Technologies, NY, USA) supplemented with 10% FBS, 5.5 mM glucose, and 100 units/mL penicillin and streptomycin at 37° C in 5% CO_2_.

### Determination of triglyceride and cholesterol content

HepG2 cells cultured in 100-mm dishes and grown to 80% confluence were cultured in serum-free medium overnight and then incubated with medium containing either normal or high glucose in the absence or presence of shizukaol D (or metformin) for the indicated times. The treated cells were lysed in RIPA buffer on ice for 45 min. The triglyceride and cholesterol content of the cell lysates were determined using a colorimetric assay kit from Sigma Aldrich and Wako as described previously [8,14].

### Transfection with small interfering RNAs

HepG2 cells were transfected with small interfering RNAs (siRNAs) (AMPKa1: 5’-GGAUCCAUCAUAUAGUUCAtt-3’, 5’-CGGGAUCAGUUAGCAACUAtt-3’) using RNiMAX (Invitrogen, Life Technologies, NY, USA). Before transfection, the medium was changed to antibiotic-free DMEM. After 24 hours of transfection, shizukaol D or metformin was added. The cells were then lysed for further analysis.

### Western blotting analysis

The cells were harvested and lysed in loading buffer. To measure the total protein concentration by Lowry method [36], the cellular proteins of the cell lysates were precipitated by using 25% TCA; and then re-dissolved in a buffer containing 2% NaOH and 0.1% SDS. Equal amounts of the protein samples (25 µg) were subjected to 8% SDS-PAGE and transferred to polyvinylidene difluoride membranes (Millipore, Bedford, MA, USA). The membranes were then blotted with primary antibodies against AMPKa, phosphor-AMPKa (Thr 172), acetyl-CoA carboxylase (ACC), phosphor-ACC (Ser 79), and beta-actin. Followed by incubation with the secondary antibody (goat anti-rabbit IgG-HRP, Santa Cruz Biotechnology, USA), the proteins were detected using a FUJIFLIM western blotting detection system (LAS-4000, FUJIFLIM, Japan) and quantified by densitometry (FUJIFLIM Multi Gauge Version 3.0).

### Mitochondrial membrane potential assay

The mitochondrial membrane potential assay was performed as described previously [14,26]. Briefly, HepG2 cells were seeded into black 96-well optical-bottom plates (Corning, Costar, USA). The cells were incubated with shizukaol D or CCCP at 37° C for 10 min, and then 100 µl of fresh medium containing 0.2 µg JC-1 was added to each well. The plates were incubated at 37° C for another 20 min, followed by washing three times with 200 µl of Krebs-Ringer phosphate HEPES buffer. The fluorescence was measured at 530 nm/580 nm (red) excitation and emission (ex/em) wavelengths and then at 485 nm/530 nm (green) ex/em wavelengths. The ratio of red to green fluorescence reflects the mitochondrial membrane potential (△ψm).

### Adenine nucleotide extraction and measurement

HepG2 cells were cultured in 60-mm dishes with shizukaol D or CCCP for the indicated period of time. The samples for cellular adenine nucleotide measurement were prepared and analyzed as previously described [37,38]. Briefly, the cells were washed with PBS buffer (140 mM NaCl, 2.7 mM KCl, 10 mM Na_2_ HPO_4_, 1.8 mM KH_2_PO_4_) and trypsinized. Next, the cells were suspended in 4% (vol/vol) perchloric acid and incubated on ice for 30 min. The pH of the lysates was adjusted to between 6 and 8 with 2 mol/L KOH and 0.3 mol/L MOPS. The precipitated salt was separated from the liquid phase by centrifugation at 13200 rpm at 4° C for 15 min. Adenine nucleotide measurements were conducted by HPLC (Agilent 1200 series) using a C18 column. The flow rate was 1.0 mL/min. The order of eluted nucleotides was ATP, ADP, and AMP. Standards (7.5 µM ATP, ADP, and AMP in ddH_2_O) were used to quantify the samples. The HPLC buffer contained 20 mM KH_2_PO_4_ and 3.5 mM K_2_HPO_4_ 3H_2_O at pH 6.1.

### Isolation of mitochondria from HepG2 cells

We isolated the mitochondria from HepG2 cells using a kit from Sigma Aldrich. 10 × 150 mm dish of cultured HepG2 cells was trypsinized, and the cells were centrifuged at 600 ×*g* for 5 min. The cells were then washed twice with ice-cold PBS, centrifuging at 600 ×*g* at 4° C for each wash. Next, 25 mL of extraction buffer A was added. The cells were incubated on ice for 15 min and homogenized for 30 strokes using a WHEATON homogenizer and then centrifuged at 600 ×*g* at 4° C for 10 min to remove the nuclei and cell debris. The supernatant was centrifuged at 11,000 ×*g* for 10 min at 4° C. The pellet was washed and centrifuged at 11,000 ×*g* for 10 min at 4° C. The resulting pellet containing the mitochondria was re-suspended in respiration medium. The protein content of the isolated mitochondria was measured using the Bradford method.

### Measurement of respiration in HepG2 cells and mitochondria isolated from HepG2 cells

Respiration measurements in both HepG2 cells and mitochondria isolated from HepG2 cells were performed using a 782 two-channel oxygen system (Strathkelvin Instruments, Motherwell, Scotland) as previously described [26]. Briefly, HepG2 cells or mitochondria were transferred to the electrode chamber and allowed to equilibrate until they attained a steady rate of oxygen consumption. Shizukaol D was then added to the chamber, and the oxygen consumption was recorded. The respiration medium used for the HepG2 cells consisted of 25 mM glucose, 1 mM pyruvate, and 2% (wt/vol) BSA in PBS, pH 7.4. For the mitochondria, the respiration medium contained 225 mM mannitol, 75 mM sucrose, 10 mM Tris-HCl, 10 mM KH_2_PO_4_, 10 mM KCl, 0.8 mM MgCl_2_, 0.1 mM EDTA, and 0.3% (wt/vol) fatty acid-free BSA, pH 7.0.

### Determination of lactate content

HepG2 cells were cultured in a 24-well plate and treated with shizukaol D or 50 µM rosiglitazone (as a positive control) in serum-free cell culture medium for 1 or 4 hours. The amount of lactate in the medium was measured using a lactate assay kit (Nanjing Jiancheng Bioengineering Institute, Nanjing, China).

### Statistics

Results were calculated as the mean ± SD, and statistical analysis was performed with SPSS. The level of significance for the difference between data sets was assessed using ANOVA followed by post-hoc test. A p-value of < 0.05 was considered significant.

## Results

### shizukaol D increases AMP-activated protein kinase (AMPK) phosphorylation

**Figure 1:**
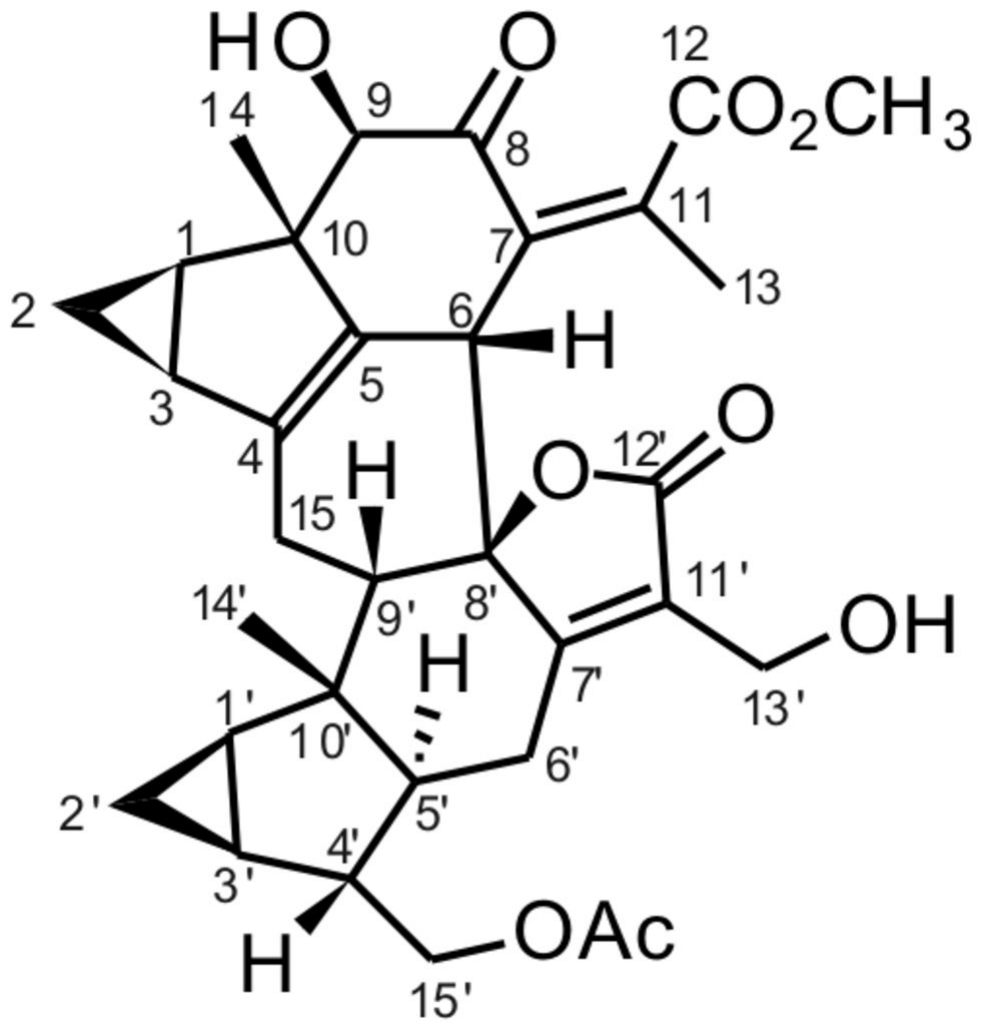
Chemical structure of shizukaol D from *Chloranthus* *japonicas* .

**Figure 2:**
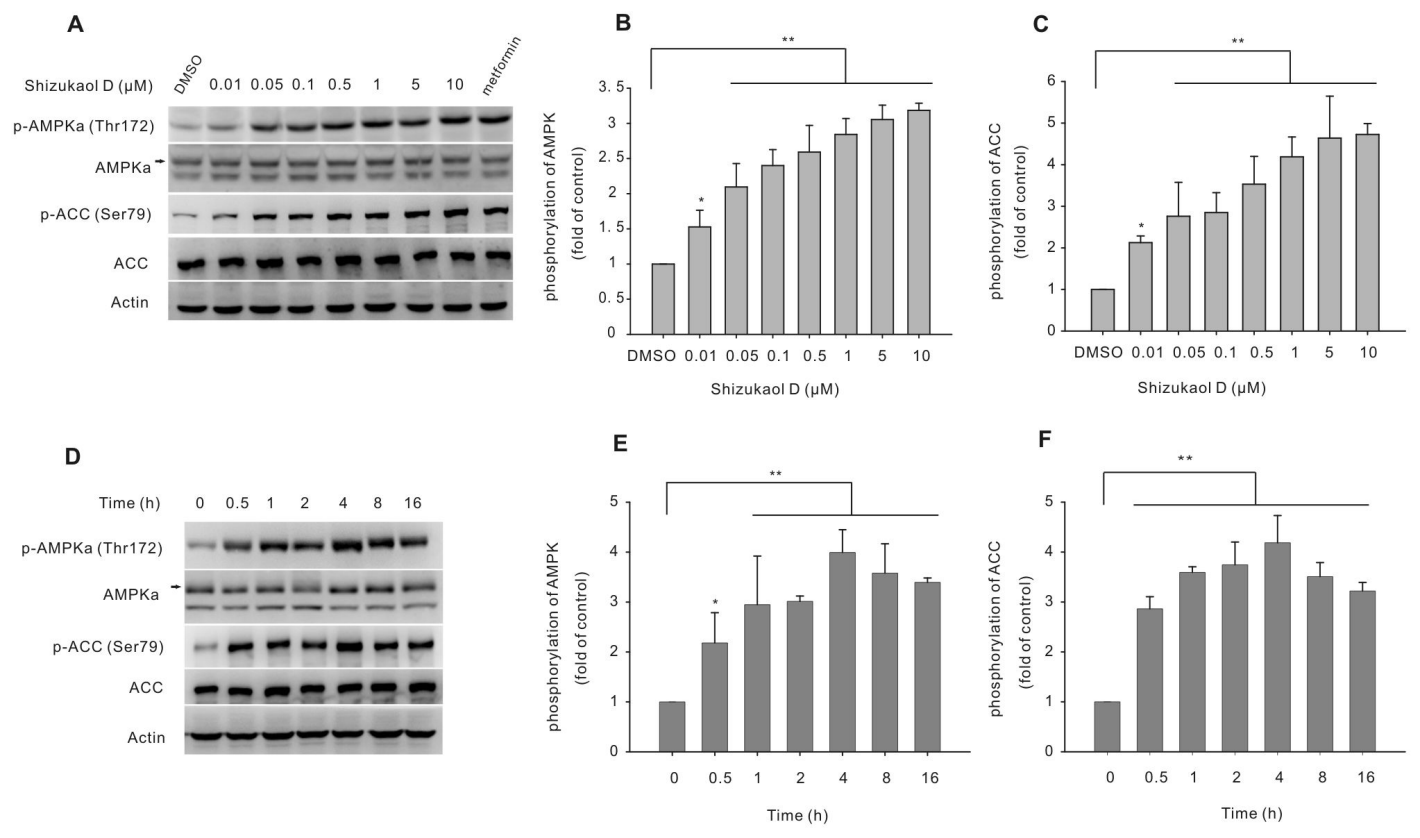
Shizukaol D increases AMPK and ACC phosphorylation in HepG2 cells. Western blotting analysis showing the levels of phosphorylated AMPK and ACC in HepG2 cells treated with shizukaol D. (A) HepG2 cells were treated with shizukaol D at the indicated concentrations for 1 h. Metformin (2 mM) was used as a positive control. (D) The cells were treated with 2 µM shizukaol D for the indicated time points. (B) (C), (E) and (F) the levels of phosphorylated AMPK and ACC were quantified from three independent experiments. *, p<0.05; **, p<0.01 compared to treatment with DMSO (one-way ANOVA).

To assess the potential effect of shizukaol D (Figure 1) on metabolism, we first analyzed the cytotoxicity of shizukaol D in HepG2 cells; we found that shizukaol D had no effect on the cell viability at various doses (maximum 50 µM) for up to 48 hours (Figure S1). We then treated HepG2 cells with shizukaol D at the indicated concentrations for 1 h, using 2 mM metformin as a positive control. The AMPK activity was analyzed by western blotting with an antibody specific for phosphorylated AMPK (Thr 172). Our results show that treatment with shizukaol D increased AMPKa phosphorylation in a dose-dependent manner (Figure 2A, B). We also assessed the phosphorylation of ACC, the downstream target of AMPK [10]. Western blotting analysis revealed that shizukaol D induced the phosphorylation of ACC at Ser 79 in a dose-dependent manner (Figure 2A, C) and we calculated that 2 µM shizukaol D induced ACC phosphorylation at a level comparable to that induced by treatment with 2 mM metformin. Finally, we treated HepG2 cells with 2 µM shizukaol D for different time points (Figure 2D, E, F).

### Shizukaol D Can Lower the Lipid Content of HepG2 Cells

**Figure 3:**
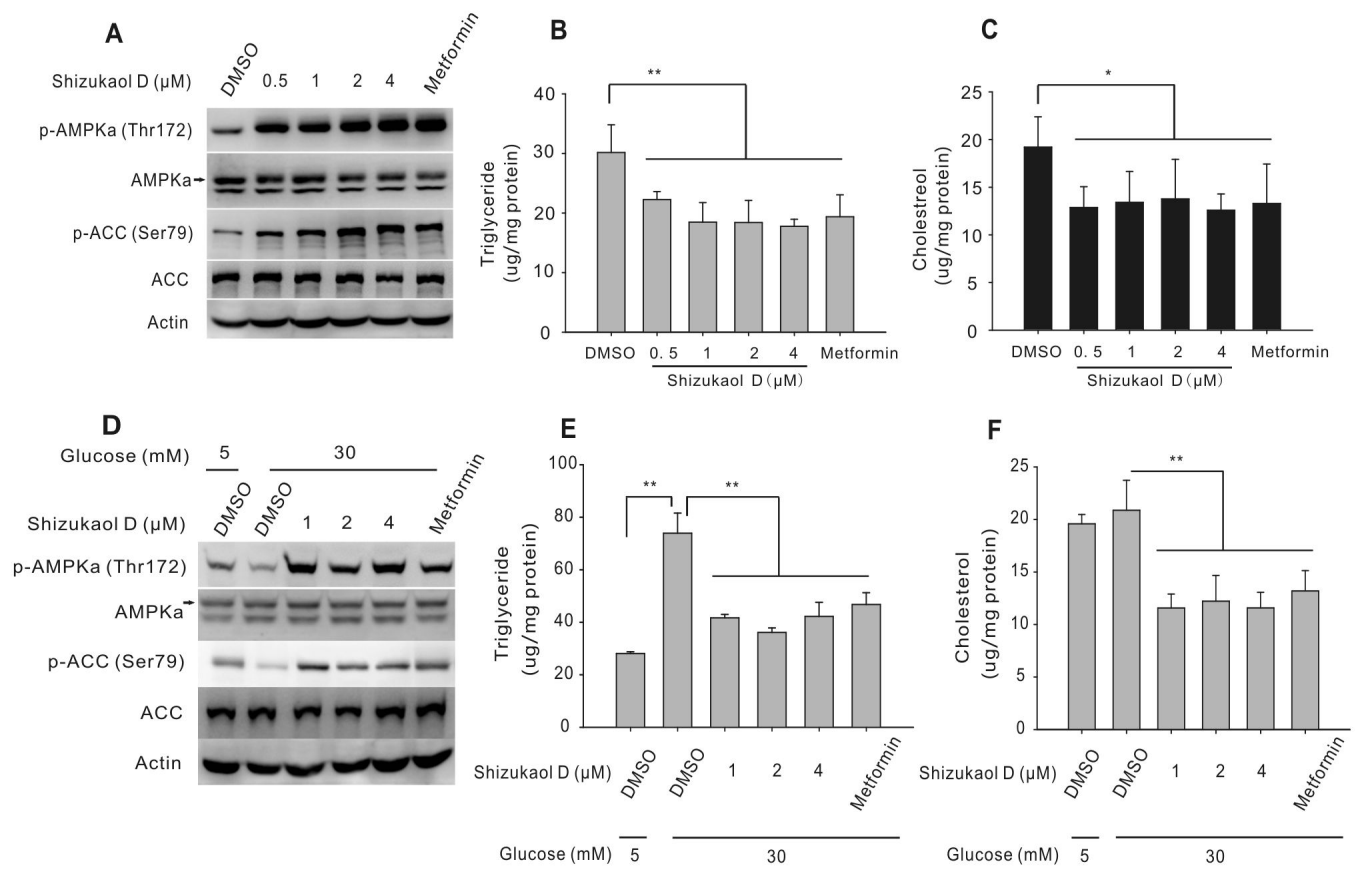
Shizukaol D inhibits lipid accumulation in HepG2 cells. HepG2 cells were starved in serum-free medium overnight and then treated with shizukaol D at the indicated concentrations for 24 h. Metformin (2 mM) was used as a positive control. Western blotting analysis showing phosphorylated AMPK and ACC (A). The triglyceride content (B) and cholesterol content (C) were measured (Results correspond to the mean ± SD of six independent experiments, statistical analysis was performed using one-way ANOVA followed by post- hoc test. *, p<0.05; **, p<0.01 versus the DMSO control). The cells were starved in serum-free medium overnight and then treated with shizukaol D at the indicated concentrations in the presence of 5.5 mM or 30 mM glucose for an additional 24 hours. The expression of AMPK and ACC was detected by western blotting (D), and the triglyceride content (E) and cholesterol content (F) were measured (graphics represent the mean ± SD from six independent experiments. *, p<0.05; **, p<0.01 versus the DMSO control).

Several studies have shown that the phosphorylation of ACC at Ser 79 leads to a reduced biosynthesis of malonyl-CoA [10,16,39], which serves as the initial substrate for fatty acid biosynthesis, and decreased carnitine palmitoyltransferase I activity, which increases mitochondrial fatty acid oxidation [17,18]. Therefore, ACC phosphorylation results in a decrease in lipid accumulation [8,9,14]. To determine whether shizukaol D can reduce lipid content, we measured the concentrations of triglycerides and cholesterol in HepG2 cells (see Materials and methods) that were starved in serum-free medium overnight and then treated with the indicated concentrations of shizukaol D for 24 h. As shown in Figure 3A, under these conditions, shizukaol D phosphorylated AMPKa (Thr172) and ACC (Ser79) as efficiently as metformin. In addition, treatment with shizukaol D significantly reduced the levels of both triglyceride and cholesterol in the HepG2 cells (Figure 3B, C).

Previous studies have shown that exposing HepG2 cells to high glucose levels (30 mM) for 24 h can induce an insulin-resistant state (Figure S2) and lipid accumulation [8,19]. To test whether shizukaol D treatment under conditions of high glucose concentrations mimics the activity of metformin, we analyzed the lipid content of shizukaol D-treated HepG2 cells exposed to medium 30 mM glucose. Our results showed that exposure to high glucose levels suppressed AMPK and ACC phosphorylation, whereas shizukaol D restored the high levels of phosphorylated AMPK and ACC, as metformin did, in the presence of high glucose levels (Figure 3D). Importantly, shizukaol D significantly reduced the high triglyceride content in HepG2 cells, which had been up-regulated due to the treatment with high glucose (Figure 3E). Interestingly, although high glucose treatment had no influence on the cholesterol level in HepG2 cells (Figure 3F), in agreement with previous studies [8,19], shizukaol D also decreased the cholesterol levels of HepG2 cells grown in high glucose medium (Figure 3F). Taken together, these results suggest that shizukaol D, like metformin, can reduce lipid accumulation in liver cells.

### The effect of shizukaol D on lipid metabolism is dependent on the AMPK-ACC signaling pathway

**Figure 4:**
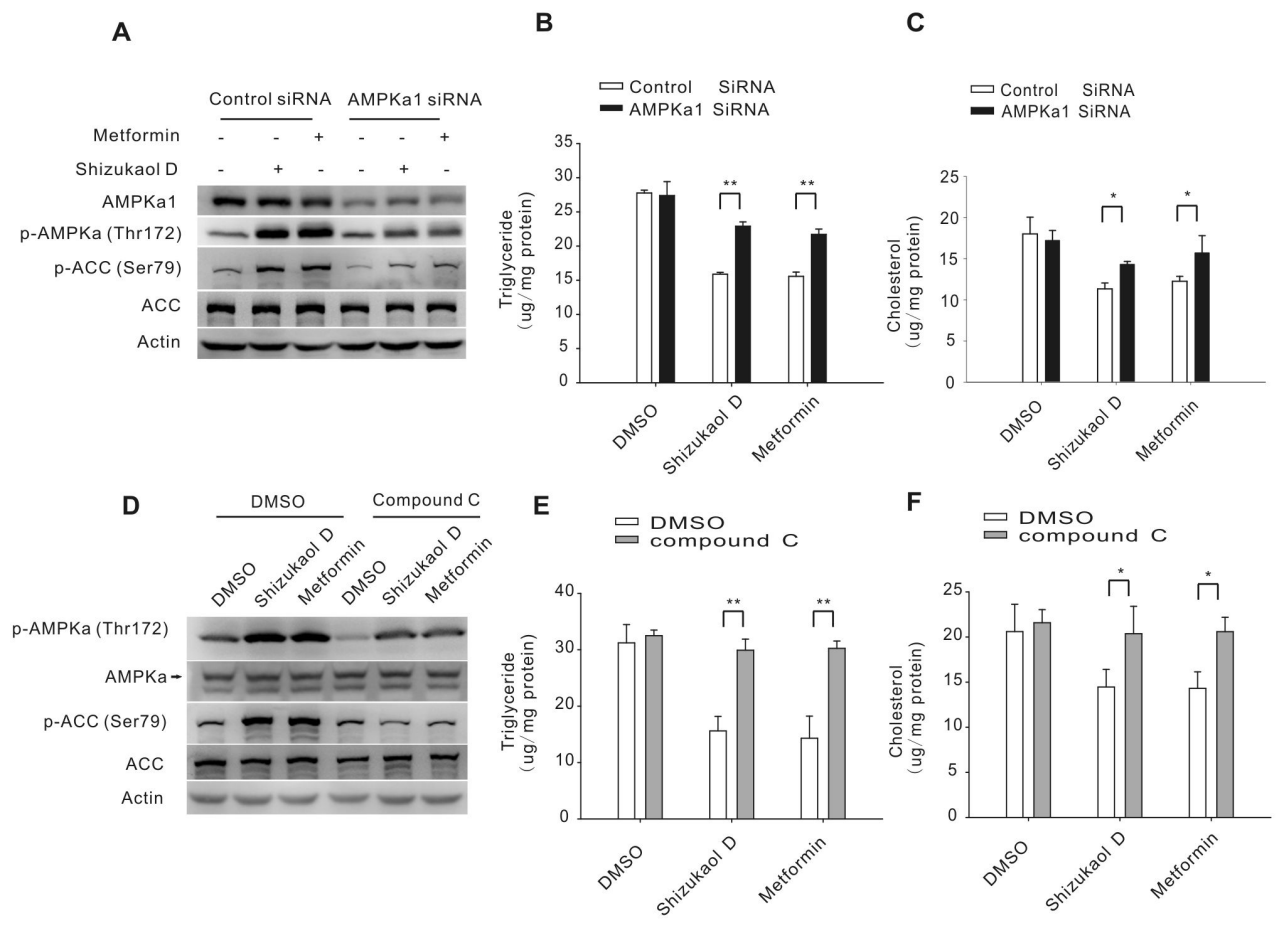
Shizukaol D inhibits lipid accumulation in HepG2 cells in an AMPK-dependent manner. HepG2 cells were transfected with AMPK siRNA or a control siRNA for 24 h followed by incubation with 2 µM shizukaol D or 2 mM metformin for an additional 24 h. AMPK and ACC phosphorylation was analyzed by western blotting (A), and the triglyceride content (B) and cholesterol content (C) were measured (n = 3). (D) The cells were pretreated with 20 µM compound C (an AMPK inhibitor) followed by treatment with 2 µM shizukaol D. AMPK and ACC phosphorylation was analyzed by western blotting (D), and the triglyceride content (E) and cholesterol content (F) were measured (n = 3). Statistical analysis was performed using two-way ANOVA followed by Tukey’ post-hoc test *, p<0.05; **, p<0.01.

To further confirm the relationship between AMPK activation and the suppression of lipid accumulation in response to treatment with shizukaol D, we inhibited AMPKa activity using an siRNA approach or with a chemical inhibitor and then detected the lipid contents of the HepG2 cells. We first transferred 50 µM siRNA into HepG2 cells to down-regulate AMPKa1 expression (Figure 4A) and then treated the cells with shizukaol D or metformin (see Materials and methods). As expected, the down-regulation of AMPKa1 expression mediated by the AMPKa1-siRNA resulted in a significant reduction in the levels phosphorylated AMPK and ACC induced by drug treatment (Figure 4A). Furthermore the siRNA treatment significantly reversed the shizukaol D-induced suppression of the triglyceride and cholesterol levels (Figure 4B, C).

Next, we inhibited AMPK with the chemical inhibitor compound C [40]. HepG2 cells were pre-treated with 20 µM compound C and then treated with 2 µM shizukaol D. Treatment of the HepG2 cells with compound C significantly inhibited the shizukaol-D-induced AMPK and ACC phosphorylation (Figure 4D). Importantly, the down-regulation of the triglyceride and cholesterol levels in HepG2 cells induced by shizukaol D was blocked by compound C (Figure 4E, F). Taken together, these results strongly support the conclusion that shizukaol D can suppress triglyceride and cholesterol levels in HepG2 cells in an AMPK-dependent manner.

### Shizukaol D decreases mitochondrial membrane potential and increases the AMP/ATP ratio

**Figure 5:**
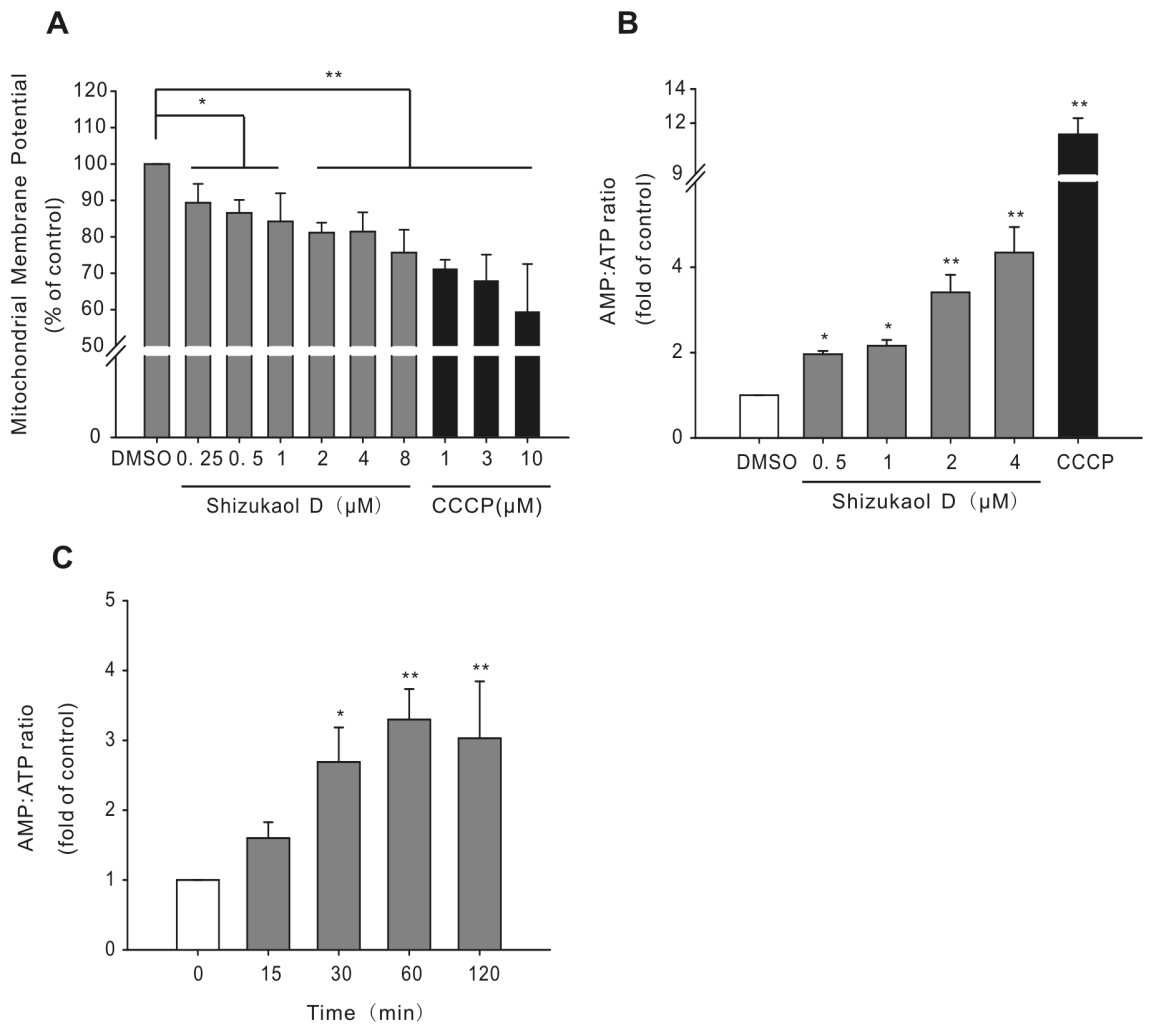
Shizukaol D inhibits the mitochondrial membrane potential and increases the AMP/ATP ratio in HepG2 cells. (A) HepG2 cells were incubated with shizukaol D for 10 min, and the mitochondrial membrane potential was measured. Treatment with CCCP was used as a positive control (n = 4). (B) HepG2 cells were treated with shizukaol D at the indicated concentrations for 1 h, and then the AMP/ATP ratio was measured (n = 3). (C) The cells were treated with 2 µM shizukaol D for the indicated time-points, and then the AMP/ATP ratio was measured (n = 3). *, p<0.05; **, p<0.01 compared to the DMSO control (one-way ANOVA).

As several studies have shown that AMPK-activating drugs such as metformin and TZDs influence mitochondrial function [24,41], we next investigated whether shizukaol D affects the mitochondrial membrane potential (△ψm) or the AMP/ATP ratio. Using a fluorescence detection assay (see Materials and methods), we observed that shizukaol D depolarized the mitochondrial membrane potential of HepG2 cells in a dose-dependent manner (Figure 5A), although the mitochondrial dysfunction induced by shizukaol D treatment was not as strong as that induced by the mitochondrial uncoupling drug CCCP (Figure 5A) [14,42,43]. ATP generation mainly occurs in mitochondria, and the inhibition of △ψm may lead to a reduction in ATP production or an increase in the AMP/ATP ratio [14]. We therefore determined the AMP/ATP ratio in HepG2 cells treated with shizukaol D by HPLC. Our results show that shizukaol D significantly increases the AMP/ATP ratio in HepG2 cells in a dose-dependent manner (Figure 5B). A time-course experiment also showed that shizukaol D increases the AMP/ATP ratio (Figure 5C). Taken together, these results suggest that shizukaol D may activate AMPK through the induction of mitochondrial dysfunction, such as the depolarization of the mitochondrial membrane and energy depletion.

### Shizukaol D inhibits cellular respiration

**Figure 6:**
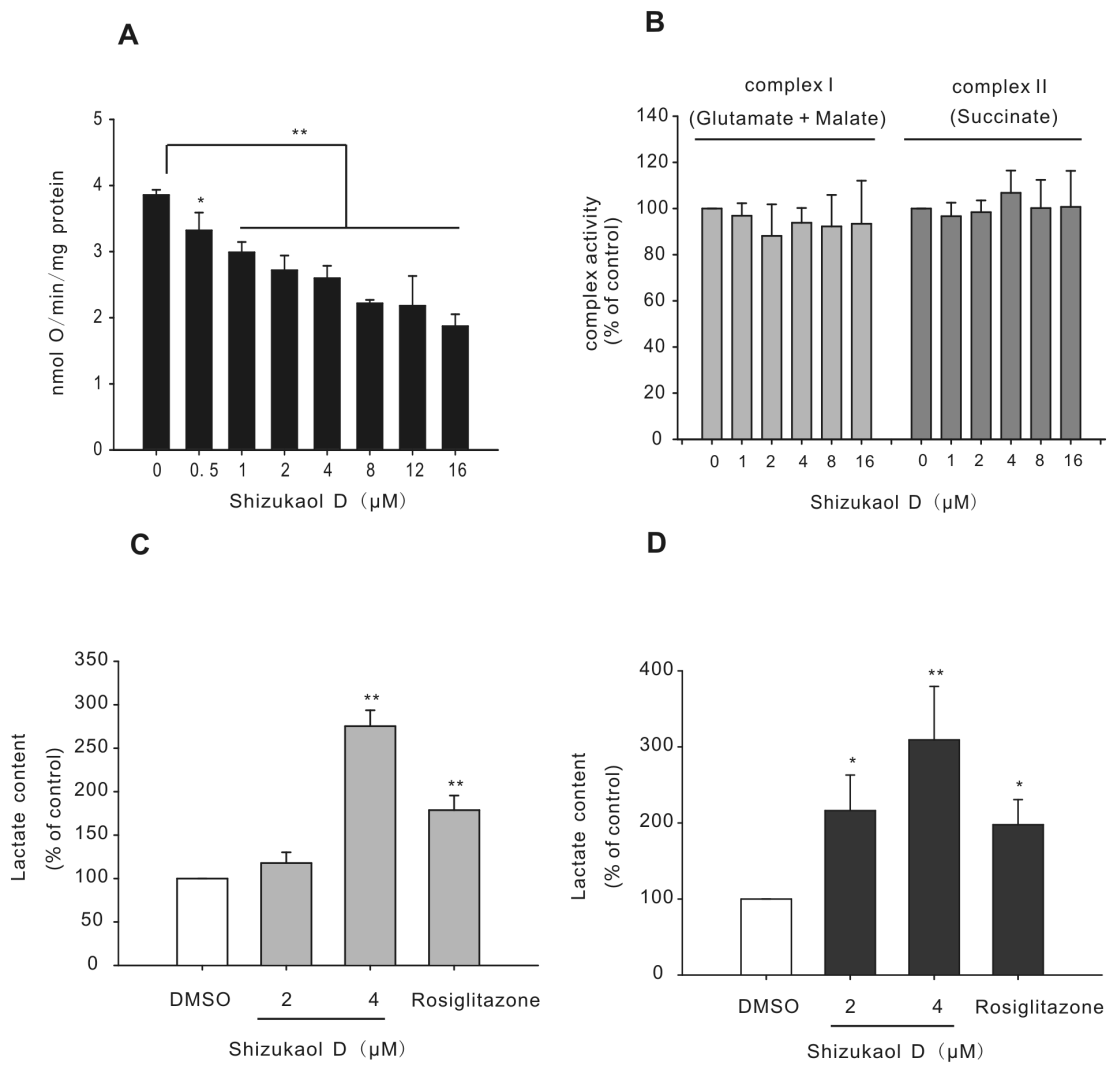
Shizukaol D inhibits cellular respiration. (A) Dose-dependent inhibition of HepG2 cell respiration by treatment with shizukaol D at the indicated concentrations (n = 4). (B) Effect of shizukaol D on the respiration of mitochondria isolated from HepG2 cells (n = 3). Shizukaol D did not inhibit mitochondrial respiration either in the presence of complex I (glutamate + malate) or complex II (succinate) substrates. (C) And (D) Lactate concentrations were measured in HepG2 cells treated with shizukaol D as indicated time (1 h and 4 h) (n = 3). *, p<0.05; **, p<0.01 compared to the DMSO control (one-way ANOVA).

To determine whether the change in the AMP/ATP ratio was due to an effect on cellular respiration (as is the case with AMPK activators such as metformin and TZDs [26,44]), we examined oxygen consumption in HepG2 cells in the presence of shizukaol D (see Materials and methods). Rosiglitazone was used as a positive control (Figure S3A) [41,45]. Treatment with shizukaol D resulted in a dose-dependent inhibition of aerobic respiration in HepG2 cells (Figure 6A). We next investigated whether shizukaol D specifically inhibits mitochondrial respiration by a mechanism similar to metformin and rosiglitazone [41,45]. ADP-stimulated respiration was analyzed in the presence of complex I (glutamate + malate) or complex II (succinate) substrates in mitochondria isolated from HepG2 cells (see Materials and methods). Rosiglitazone was used as a control for the specific inhibition of complex I (Figure S3B) [41]. We observed that shizukaol D did not inhibit mitochondrial respiration using either substrate (Figure 6B).

Previous reports have shown an elevation in anaerobic respiration to compensate for the suppression of aerobic respiration [14,26,46]. Therefore, we analyzed whether shizukaol D modulates lactate release, which is a marker of cellular anaerobic respiration. HepG2 cells were treated with shizukaol D for 1 h and 4 h, and the lactate concentration was measured (see Materials and methods). Elevated levels of lactate were found in HepG2 cells treated with shizukaol D (Figure 6C, D). This finding suggests that the suppression of aerobic respiration induced by shizukaol D results in the up-regulation of anaerobic respiration to meet the energy requirement of the cells.

## Discussion

In this study, we have shown that shizukaol D reduces triglyceride and cholesterol levels in HepG2 cells grown at a normal concentration of glucose (5.5 mM; Figure 3B, C). The reduction of lipid content induced by shizukaol D may be a result of ACC phosphorylation and/or the activity of other enzymes such as fatty acid synthase (FAS), sterol regulatory element-binding protein 1 (SREBP1), and 3-hydroxy-3-methylglutarl-coenzyme A reductase [47–49]. However, neither shizukaol D nor metformin could alter cellular palmitic acids content after 12 hours incubation (Figure S4). The exposure of HepG2 cells to high glucose (30 mM) for 24 h induces an insulin-resistant state (Figure S2) [8,50,51] and a decrease in both AMPK and ACC phosphorylation (Figure 3D) [8,52]. In addition, our results agree with previously published studies showing that high glucose concentrations dramatically increase the triglyceride content in HepG2 cells but do not dramatically increase the cholesterol content [8,19] (Figure 3E, F). Furthermore, shizukaol D restored the levels of both AMPK and ACC phosphorylation that had been reduced by high glucose concentrations (Figure 3D). Because treatment with shizukaol D inhibits the triglyceride and cholesterol content in HepG2 cells in the presence of either low glucose (Figure 3B, C) or high glucose (Figure 3E, F), we propose that shizukaol D can lower the lipid content in HepG2 cells in both normal and insulin-resistant states.

To confirm the significance of AMPK in the activity of shizukaol D, we inhibited AMPK using an AMPKa1 siRNA and the AMPK inhibitor compound C. AMPKa1 siRNA knocks down the expression of AMPKa1, an important subunit of AMPK that has a phosphorylation site on a conserved loop at Thr 172. A previous study showed that AMPKa1 knockdown inhibited the ability of metformin to activate AMPK and down-regulate lipid content [8]. Compound C causes a remarkable inhibition of AMPK activity [40]. Here, we observed that both AMPKa1 siRNA and compound C decreased the shizukaol D-mediated phosphorylation of AMPK and abrogated the ability of shizukaol D to reduce lipid levels. This finding suggests that the modulation of lipid metabolism by shizukaol D is largely dependent on the AMPK-ACC signaling pathway.

A number of AMPK activators, such as metformin, TZDs, and berberine, are known to generate mitochondrial dysfunction in cells [41,45]. Here, we show that shizukaol D also decreased the mitochondrial membrane potential of HepG2 cells (Figure 5A), although we did not detect the expression of any apoptotic markers in response to the drug treatment (data not shown).

AMPK activation is a direct result of alterations in the AMP/ATP ratio [44,53–55]. Here, we found that treatment with shizukaol D increased the AMP/ATP ratio (Figure 5B, C). Furthermore, shizukaol D inhibited cellular respiration, similar to metformin and rosiglitazone (Figure 6A) [41]. We further investigated whether shizukaol D inhibits respiration in mitochondria isolated from HepG2 cells (the mitochondrial purity was approximately 60-70%, as shown in Figure S5) [56]. Surprisingly, we found that shizukaol D did not inhibit mitochondrial respiration using either complex I (glutamate and malate) or complex II (succinate) (Figure 6B). This finding suggests that other factor(s) may regulate aerobic respiration, such as the supply of electron donors (e.g., NADH) [14,54,57]. The inhibition of these factors may lead to the inhibition of aerobic respiration in cells, which would not be apparent in assays measuring the respiration of isolated mitochondria. Previous reports have shown that indomethacin, an anti-inflammatory drug, suppresses glucose oxidation without affecting pyruvate oxidation in mitochondria [58,59]. Furthermore, the compound C1 inhibits aerobic respiration but does not affect the activity of complex I or complex II in mitochondrial respiration [14]. Our findings highlight the potential value of shizukaol D as a promising compound for the treatment of metabolic diseases by activating AMPK.

## Supporting Information

Figure S1 **Survival analysis of shizukaol D-treated HepG2 cells.** The viability of HepG2 cells treated with shizukaol D at the indicated concentrations for different time-points was analyzed by MTT assay. The results were normalized to the viability of DMSO-treated cells, which was set as 100%. Error bars represent the SD. from three independent experiments.
(TIF)

Figure S2 **High glucose medium-induced insulin resistance of HepG2 cells.** After incubation in normal (5 mM) or high (30 mM) glucose medium for 24 hours, HepG2 cells were incubated with 100 nM insulin for 10 min. Two components of the insulin signaling pathway were detected by western blotting.
(TIF)

Figure S3 **Analysis of respiration in HepG2 cells and mitochondria isolated from HepG2 cells.** (A) Rosiglitazone was set as control in HepG2 cellular respiration analysis (n=4). (B) Analysis of ADP-stimulated respiration in the presence of complex I (glutamate + malate) or complex II (succinate) substrates in mitochondria isolated from HepG2 cells. Rosiglitazone was used as specific inhibitor for complex I (n=3). *, p<0.05; **, p<0.01 versus control (one-way ANOVA).
(TIF)

Figure S4 **Shizukaol D doesn’t alter the free fatty acids (palmitic acid) in HepG2 cells.** HepG2 cells were starved in serum-free DMEM overnight and incubated with shizukaol D for 12 hours. The cells were then lysed in chloroform (1% Triton-X 100) for 30 min, and the level of fatty acids (palmitic acid) was detected (n = 3).
(TIF)

Figure S5 **Assessment of mitochondrial purity by western blotting.** Mitochondria were isolated from HepG2 cells. The purity was then assayed using a panel of marker proteins including Cytochrome C, Porin (mitochondria), Lamin B (Nucleus), HSP90 (cytosol), and Grp 78 (endoplasmic reticulum). PM represents isolated mitochondria; CT is cell lysates after homogenized.
(TIF)
