# Supplementary figures and images for: Shizukaol D Isolated from Chloranthus japonicas Inhibits AMPK-Dependent Lipid Content in Hepatic Cells by Inducing Mitochondrial Dysfunction
Source: PLoS One. 2013 Aug 14;8(8):e73527. doi: 10.1371/journal.pone.0073527 (PMC3743771; doi:10.1371/journal.pone.0073527)

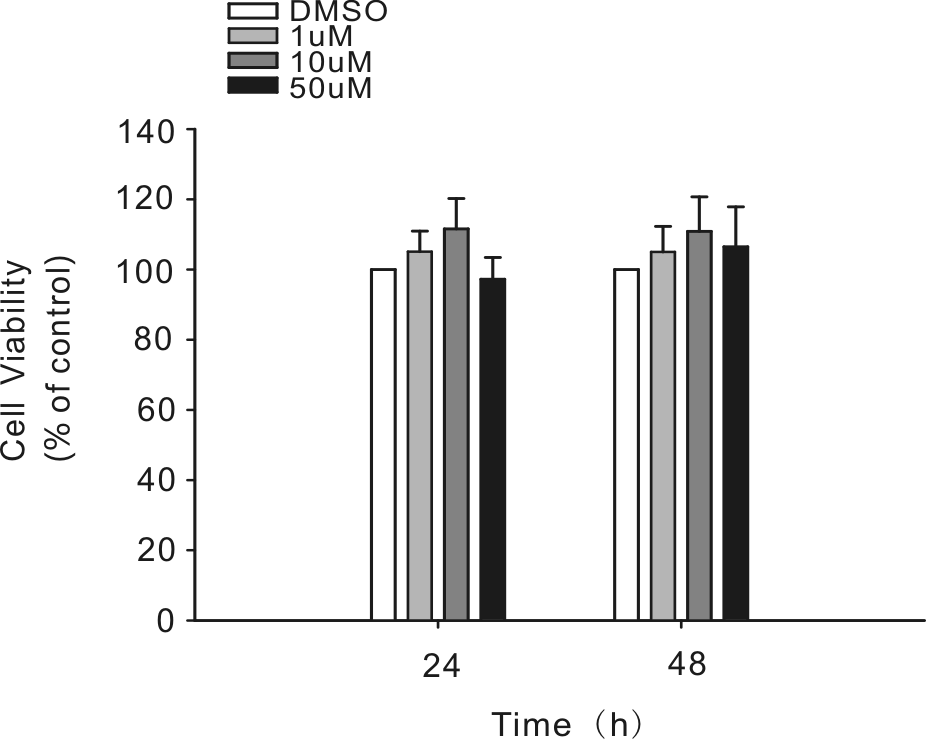

Supplement: Figure S1 — Survival analysis of shizukaol D-treated HepG2 cells. The viability of HepG2 cells treated with shizukaol D at the indicated concentrations for different time-points was analyzed by MTT assay. The results were normalized to the viability of DMSO-treated cells, which was set as 100%. Error bars represent the SD. from three independent experiments. (TIF) [file pone.0073527.s001.tif]

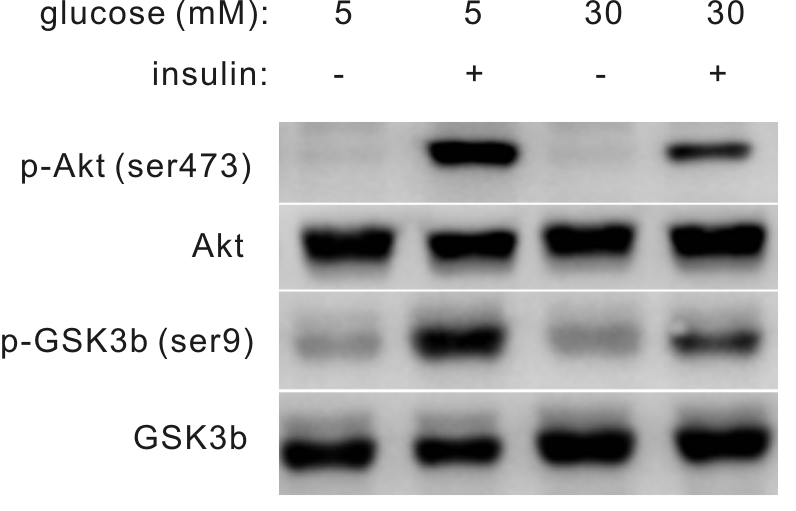

Supplement: Figure S2 — High glucose medium-induced insulin resistance of HepG2 cells. After incubation in normal (5 mM) or high (30 mM) glucose medium for 24 hours, HepG2 cells were incubated with 100 nM insulin for 10 min. Two components of the insulin signaling pathway were detected by western blotting. (TIF) [file pone.0073527.s002.tif]

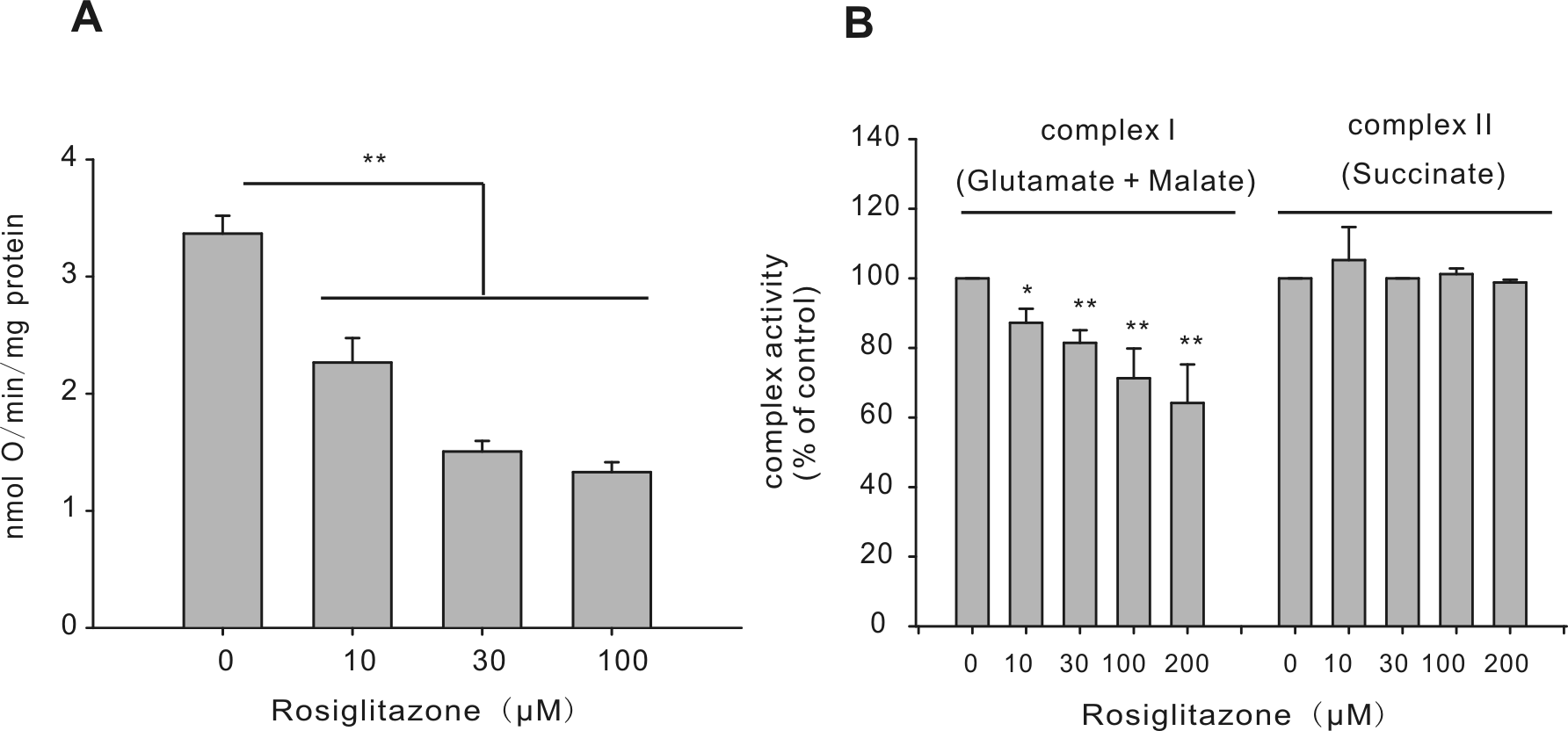

Supplement: Figure S3 — Analysis of respiration in HepG2 cells and mitochondria isolated from HepG2 cells. (A) Rosiglitazone was set as control in HepG2 cellular respiration analysis (n=4). (B) Analysis of ADP-stimulated respiration in the presence of complex I (glutamate + malate) or complex II (succinate) substrates in mitochondria isolated from HepG2 cells. Rosiglitazone was used as specific inhibitor for complex I (n=3). *, p<0.05; **, p<0.01 versus control (one-way ANOVA). (TIF) [file pone.0073527.s003.tif]

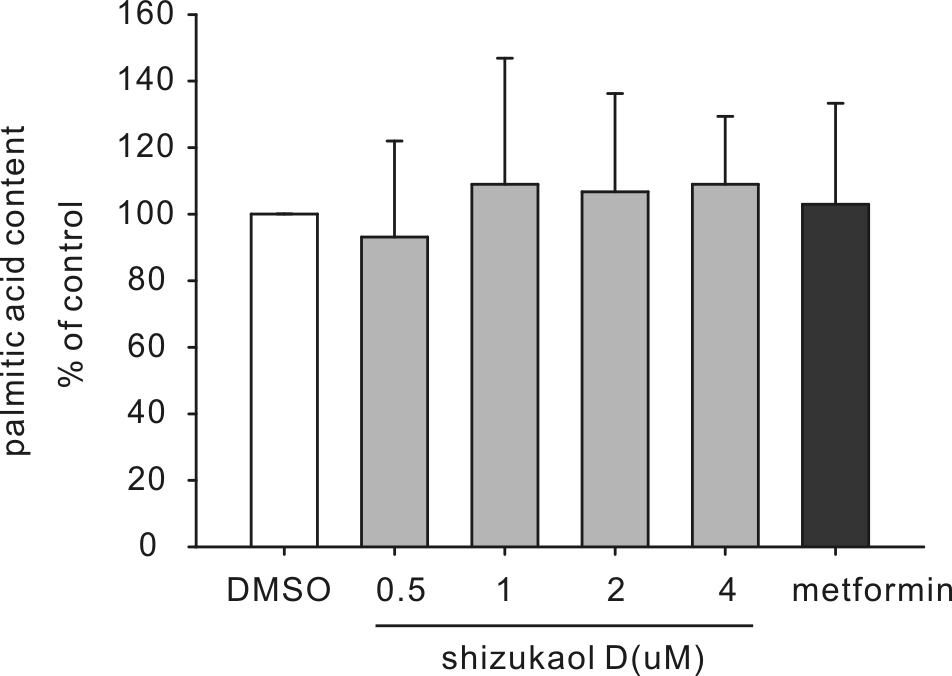

Supplement: Figure S4 — Shizukaol D doesn’t alter the free fatty acids (palmitic acid) in HepG2 cells. HepG2 cells were starved in serum-free DMEM overnight and incubated with shizukaol D for 12 hours. The cells were then lysed in chloroform (1% Triton-X 100) for 30 min, and the level of fatty acids (palmitic acid) was detected (n = 3). (TIF) [file pone.0073527.s004.tif]

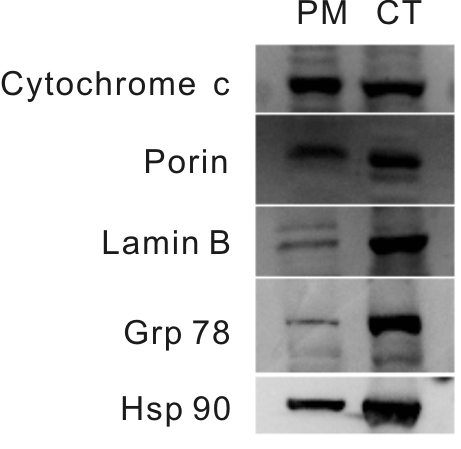

Supplement: Figure S5 — Assessment of mitochondrial purity by western blotting. Mitochondria were isolated from HepG2 cells. The purity was then assayed using a panel of marker proteins including Cytochrome C, Porin (mitochondria), Lamin B (Nucleus), HSP90 (cytosol), and Grp 78 (endoplasmic reticulum). PM represents isolated mitochondria; CT is cell lysates after homogenized. (TIF) [file pone.0073527.s005.tif]
